# Separate the Operative from the Mechanical

**Published:** 1889-08

**Authors:** W. W. Allport

**Affiliations:** Chicago.


ARTICLE IV.

### SEPARATE THE OPERATIVE FROM THE MECHANICAL.

DR. W. W. ALLPORT, CHICAGO.

(Extract of an address before the American Academy of Dental Science).

That mechanical dentistry should have very largely fallen into the hands of an inferior class of practitioners will hardly be wondered at by those who have watched the history of that branch of the practice. Up to about thirty years ago, the mechanical department of the practice required a practical knowledge of selecting and compounding the materials of which the teeth were made; the hand and the eye of an artist were requisite to give them form and color; the management of heat in baking them; a knowledge of the nature of precious metals and skill in working them; and a high order of mechanical talents in applying intricate mechanical laws in fitting and rendering useful the different forms of plates, together with mechanical and artistic skill in so adjusting the substitutes as to subserve the purposes for which they were intended. Since then, the manufacture of artificial teeth has become a distinct business, and they are now simply articles of commerce, bought by the piece, set, or thousand; and to such perfection has this branch of manufactures been carried, that few dentists now think of making the teeth they use. Plates of precious metal, requiring mechanical skill of a high order to manipulate, have in a large majority of cases, been substituted by plates cast from baser metals, or by rubber vulcanized in molds, these requiring neither a high degree of judgment nor mechanical skill to accomplish results tolerably well limited by the properties of the material used.

As a consequence, therefore, of these conditions, the surgical branch of dentistry, which, when practiced by competent men, allies it to medical science, has been constantly on the advance; while that which is devoted to the setting of artificial teeth has, in the last few years, been steadily retrograding and becoming more and more a trade. And so simple are the modes of attaining tolerable mechanical results with the methods now usually employed in this department, that a high order of appropriate talent is, at the present time, seldom found devoting much time to it. By this I would not be understood as saying that this latter department does not need improving; for when viewed as an art, he who has but moderate ideas of symmetry or harmony of expression and color is constantly pained by the lack of that artistic selection and arrangement of artificial teeth which serves to restore to the face the shape and expression left on it by the Creator, the absence of which in artificial dentures stamps him who should be an artist, an artisan—a mere mechanic—a libeller of the soul—a deformer of the human face divine.

At the present time there are about twelve thousand practicing dentists in the United States, about two thousand of whom are regular or honorary graduates of either dental or medical schools. To offset the unworthy graduates by those practitioners not graduates, who, by study and exertion have earned a deserved reputation and position in practice, would, I think, form a fair estimate of the really competent practitioners of scientific dentistry in the United States at the present day—making the number of the really qualified about one sixth of the entire number. Assuming, therefore, that the one-sixth, as are the members of your Academy, sufficiently advanced in the knowledge of medical science to entitle them to the right to be regarded as special practitioners of medicine, it can hardly be expected that dentistry, as a proffession, when taken as a whole, would or ought to be so regarded by medical men.

This claim, while being justly made by some, and freely acknowledged as to a portion of dental practitioners, individually has, not without cause, I think, been denied to the profession at large, The tastes, habits, and acquirements of the two classes of dental practitioners are as divergent as are the characters of true science and mechanism; the practice of the one being established on a medical basis, while the other relates only to a mechanical art. The practice of either branch, it is true, involves a limited knowledge of the other; but it is necessary either for the surgical practitioner to be a practical mechanic, or the mechanician on artificial work to understand the rationale of medical treatment, or to be an operator. In fact, the practice of both by the same individual prevents the highest development of either as would the time spent in the manufacture of artificial legs by the surgeon, or the compounding, baking and coloring of artificial eyes by the ophthalmologist, serve but to retard the higher development of their specialties; or an attempt by the maker of limbs, eyes or optical instruments to practice general surgery or the treatment of the eye, but degrade his own proper art.

As I have before stated, the yoking together of the two callings seemed to be a necessity of the condition of the practice, at the time they were joined, and has resulted in great good. But the development of the practice has now brought us to a point where it is clear a new departure should be taken, the co-partnership dissolved, and each department followed as a distinct and separate calling. Neither in private offices nor in our colleges should the two be taught as one, nor should the term "dentist" be retained in our nomenclature.

The true mission of medical science is to preserve or restore health and save life and limb, not to make or have to do with the making of artificial substitutes any further than as they shall be made directly useful in subserving these purposes. Wig-making and the manufacture of artificial limbs and eyes are useful and respectable callings, and, when properly pursued, require a good degree not only of mechanical skill, but also of artistic taste; and as well, almost, might the making of these be taught in the medical college as the making of sets of artificial teeth form part of the curriculum in a medical specialty. Tbe long association of operative with mechanical dentistry will make it somewhat more difficult, at first, to disconnect them in the minds of the public, than in practice, as separate callings; but no professional act would be so directly instrumental in accomplishing this result as to drop mechanical dentistry from the curriculum of our colleges, and save the time usually devoted to the teaching and practical work in the manufacturing and mounting of artificial teeth, and to other laboratory work, and employ it in giving broader and more comprehensive instruction in the science of medicine in these schools, or else, to incorporate them with the regular colleges of medicine by the establishment of appropriate chairs and infirmaries for clinical teaching.

Let dental mechanics be otherwise taught as a high mechanical art, and the calling fixed in the mind of the public as such, and, in a few years, a patient would as soon go to the maker of artificial legs for advice or treatment in conservative surgery, or regarding amputation, as to the *dentican* or *dentificier*, for advice or services in the saving of his natural teeth, or their extraction.

To drop the teaching of mechanical dentistry in private offices and in our colleges would, in a few years, permanently divide the practice, and very soon each town, of any considerable size, would have one or more of these practitioners who, by relying entirely on success in this calling for support and becoming personally responsible for what they did, would seek to redeem and elevate this particular art to the highest attainable, thereby enhancing the respectability and usefulness of their calling. And the *dentologist* would, by the broad and comprehensive teachings of medicine, become more thoroughly grounded in its science, and be better qualified to take his rank with the other medically recognized specialists. With this thorough groundwork laid, he would not only be better prepared to treat from a medical standpoint the diseases belonging to his province, but also to grapple successfully by general treatment with those hidden and hither ill understood influences which serve to prevent perfect dental development, and also to counteract those pathological conditions which act as causes of disease in the teeth and tend to break down their tissues.

With the development of this higher mission of our profession there will be no occasion for the spectacle of dentology, with the grimace and shuffle of the mendicant approaching the gates of the medical profession, and, with downcast eyes, begging a crumb of recognition. But with the accomplished separation of the two callings, heretofore combined in our practice, dentology, enriched by the experience and the special literature of the last half century, and the foundation of its practice laid exclusively in the science of medicine, rather than divided between that and a trade, the incongruity of the past will, in a few years, disappear. By deriving its nourishment from the body of which it is a branch, it will become more and more assimilated to the science and the practice of medicine, and, without demand or the asking, there will, both by the public and the medical faculty, be accorded, not to individual practitioners, but to the branch, a full and cordial recognition as a specialty in medicine, which will attract more generally to its ranks, as to an agreeable and useful field of labor, men of earnestness, ability, and culture, the peers of any in an honorable profession.

